# Innovative method integrates play fairway analysis supported with GIS and seismic modeling for geothermal potential evaluation in a basement reservoir

**DOI:** 10.1038/s41598-024-79943-6

**Published:** 2025-01-08

**Authors:** Mohamed Ayed Elbalawy, Mohamed Balash, Mohamed Hamdy Eid, Ernő Takács, Felicitász Velledits

**Affiliations:** 1 Faculty of Earth and Environmental Sciences and Engineering, Institute of Exploration Geosciences, University of Miskolc, Miskolc, Hungary; 2 Faculty of Science, Geophysics Department, Ain Shams University, Cairo, Egypt; 3 Faculty of Sciences, Geology Department, South Valley University, Qena, Egypt; 4 Faculty of Science, Geology Department, Beni-Suef University, Beni-Suef, 65211 Egypt; 5 Supervisory Authority for Regulatory Affairs, Budapest, Hungary

**Keywords:** Geothermal energy, GPFA, Risk Assessment, Sustainable energy, Pannonian basin, Geothermal energy, Geology, Geophysics

## Abstract

The growing demand for clean and sustainable energy sources has prompted the investigation of numerous renewable and ecologically friendly options. Among these, geothermal energy is particularly noteworthy because of its widespread availability, compact size, and consistent, weather-independent power production. A geothermal play fairway analysis (GPFA) model was created for the study area, which is located in Békés county, southeastern Hungary. The GPFA model approach in the current study is the first model developed in Hungary to achieve three main goals. firstly, to quantitatively assess the geothermal potential, secondly, to identify the most favorable areas for geothermal exploration and development, and thirdly, to evaluate the corresponding risk levels in the study area. The study focuses on identifying and assessing three main risk components associated with exploitable geothermal systems in the study area. The risk parameters consist of the heat source, reservoir fracture permeability, and seal. Advanced 3D seismic interpretation, geographic information system (GIS), and 3DHIP (heat in place) calculator techniques are used to evaluate subsurface structural and thermal models. Two phases of seismic interpretation are used; conventional interpretation phase focused on conventional seismic data interpretation and advanced attribute generation phase where various seismic attribute cube volumes are generated. Common Risk Segment Maps (CRS) for each risk parameter are created by combining data from all the elements contributing to that risk using GIS toolbox. The resulted CRS maps of the study area three risk parameters are summed to produce a Composite Common Risk Segment Map (CCRS) map. Based on the constructed CCRS map and the developed GPFA model, the study area holds valuable untapped geothermal potential, poses varying risk levels associated with geothermal exploration and development. The amount of risk resulting from the three risk components is not equal, and the reservoir fracture permeability is the main risk factor. The GPFA model is successfully narrowed down an expansive exploration area of around 350 km^2^ to just 4 highly promising targets with high geothermal favorability and low risk as future drilling targets. The constructed 3D thermal capacity model indicates that the average heat content in the study area is estimated to be 65,450 Petajoules per square kilometer (PJ/km^2^), with a recoverable heat energy of 6090 megawatt thermal per square kilometer (MWth/km^2^). The recoverable heat for the four selected targets is estimated under different production scenarios: a 30-year plan, a 20-year plan, and a 10-year plan and it ranges from 7.5 to 32 MWth/km^2^, 11 to 48 MWth/km2, 22.2 to 96.8 MWth/km^2^ respectively. The findings of this study have made important contributions to the field of geothermal exploration approaches and offer valuable insights for making well-informed decisions about sustainable energy development in the study area.

**Supplementary Information:**

The online version contains supplementary material available at 10.1038/s41598-024-79943-6.

## Introduction

The growing demand for clean and sustainable energy sources has prompted the investigation of numerous renewable and ecologically friendly options. Among these, geothermal energy is particularly noteworthy because of its widespread availability, compact size, and consistent, weather-independent power production^1^. It is also a vital tool in the fight against climate change because it has zero carbon emissions^2^. The use and adoption of geothermal energy have progressed relatively slowly, despite its enormous potential^3^. This is mostly because conventional geothermal well drilling is difficult, expensive, and fraught with risk. It is also frequently restricted to particular regions with active conventional hydrothermal and volcanic activity^4^. Geothermal technology has advanced significantly because of the increased demand for clean energy worldwide. Twenty countries generate 73.7 Terawatt hours (TWh) of electricity annually from geothermal energy^5^. Furthermore, geothermal energy is used for direct heating and cooling; it is used in about 70 countries worldwide and produces roughly 163 TWh annually^6^. The global adoption of geothermal energy has expanded from 58 countries in 2000 to 88 countries in 2020. In 2019, the estimated installed thermal power for direct utilization was 107,727 MWth, which represents a 52.0% increase compared to the data from 2015. This growth occurred at a compound annual rate of 8.73%. The annual thermal energy consumption is 1,020,887 terajoules (283,580 gigawatt hours), which represents a 72.3% increase compared to the year 2015. This growth has been consistent, with an annual compound rate of 11.5%^7^. Geothermal power generation capacity increased by a significant 27% (3.65 Gigawatt) between 2015 and 2020, with significant growth taking place in Turkey, Indonesia, the USA, and Kenya^8^. According to forecasting, growth will continue, with estimates for direct thermal use reaching 3300–3800 TWh/year and electricity generation reaching 800–1300 TWh by 2050^9^.

Hungary’s location in the Pannonian Basin offers a wealth of geothermal potential. The Middle Miocene (17.5–12.5 Ma) lithospheric extension in the Pannonian Basin has led to significant heat flow density, causing the lithosphere to become thinner and the asthenosphere to rise^10–12^. The average heat flow density in Hungary is 90 mW/m^2^, with fluctuations ranging from 30 mW/m^2^ to 120 mW/m^2^. The mean value of 90 mW/m^2^ exceeds the average of 65 mW/m^2^for continental heat flow density^13,14^. The eastern and southern parts of the country have high heat flow values due to buried basement highs, while the recharge areas of karstic flow systems have low values. In the sedimentary basins, the thickness of the Neogene and Quaternary sediments can reach 5–7 km. The heat flow in these areas is slightly lower than the average value of 80–90 mW/m^2^. These sedimentary basins are highly regarded as the primary thermal water aquifers in Hungary, widely utilized for district heating and greenhouse heating purposes. The buried basement highs have a significant heat flow and typically exceed the average value (100–120 mW/m^2^), making them attractive locations for geothermal exploration applications^13^. Geothermal energy is being extensively investigated in Hungary as several authors have provided comprehensive insights into geothermal energy exploration, covering various aspects such as the history of geothermal energy in Hungary, the current status of geothermal energy production and utilization, an analysis of geothermal conditions, risk management in geothermal projects, and updates on the country’s geothermal sector^15–17,7,18^. Several authors have discussed the geological and thermal assessment of known reservoirs or abandoned oil wells, such as the works by^19–21^. Based on the previous literatures, it is clear that there exists a notable research gap in the investigation and quantitative evaluation in areas where there are no surface geothermal manifestations or any previous indications (referred to as blind geothermal systems) especially deep basement targets. Furthermore, it is imperative to precisely assess the risks associated with the exploration and development of these systems. This gap will be bridged through the used methodology in this study.

Play Fairway Analysis (PFA) is an exploration approach that has been originated by experts in the petroleum industry. This process entails the integration of regional or basin-scale data to systematically identify promising patterns for exploration. Data is combined to pinpoint the highest probability of success of the plays. The approach provided by PFA is recognized for its exceptional technical rigor, exceeding that of conventional exploration approaches. This approach allows for informed decision-making, even in scenarios with limited or insufficient data^22,23^. PFA is widely recognized as a proven methodology in the petroleum industry, while it is still gaining traction as an exploration technique in the geothermal industry. The geothermal industry has made significant progress, moving beyond drilling conventional visible geothermal systems towards currently exploring blind systems within known or inferred geothermal trends. Nevertheless, PFA has not been widely embraced by the geothermal industry. An initiative supported by the U.S. Department of Energy between 2014 and 2021 has sparked considerable interest in this application^24^. The research conducted by many authors like^23–28^showcases that the PFA approach could be valuable in uncovering hidden or concealed geothermal systems. However, adapting the PFA to produce valuable outcomes for different geothermal settings and a measurable return on investment is a significant challenge^27^.

The study aims to achieve three primary objectives: firstly, to conduct a quantitative assessment of the geothermal potential in terms of thermal energy stored within the reservoir in Petajoules per square kilometer (PJ/km^2^), and the recoverable energy potential in megawatt thermal per square kilometer (MWth/km^2^) using the 3DHIP calculator. secondly, to identify the most favorable areas for geothermal exploration and development through utilizing the final combined Composite Common Risk (CCRS) map; and thirdly, to evaluate the associated risk levels in the study area using the developed GPFA of the study area. The geothermal play fairway analysis (GPFA) model will be constructed by gathering and examining all the relevant information for the study area from a variety of sources, covering both publicly accessible and private data. Our study is focused on identifying and assessing three critical risk parameters for exploitable geothermal systems in the study area: heat source, reservoir fracture permeability, and seal. The heat source is a crucial factor that must be carefully assessed and evaluated, as it is essential for the proper functioning of a geothermal system. The reservoir fracture permeability serves as the conduits that allow fluids to flow, carrying heat to the surface. The seal effectively insulates the reservoirs, ensuring that no venting occurs to the surface. The seal poses a significant risk in the Pannonian basin due to the vertical connection of the Upper Pannonian aquifer system through semipermeable layers. The pumping tests indicate a hydraulic connection between the shallower cold-water aquifers and the deeper thermal aquifers^29^. The compilation for heat source includes data such as heat flow maps and estimates of reservoir temperature. The fracture reservoir permeability is determined by analyzing stress orientations and magnitudes, fracture attribute analysis, inversion results, and published fault maps. Data for the seal includes the spatial coverage and vertical thickness of the impermeable upper basement marl deposits. Common Risk Segment (CRS) maps for each risk parameter are created by combining data from all the elements contributing to that source of risk parameter. The maps generated from these three components are utilized to create the ultimate predictive CCRS map which represents the GPFA model of the study area. Using geothermal PFA approach, our primary focus is on minimizing risk in exploration and directing our efforts toward areas with a higher likelihood of success. Our intention is to fill the research gap by presenting this innovative study in Hungary. In addition, this study incorporates novel input data for the initial implementation of the PFA approach. This novel data encompassed colored inversion as a sign of fracturing, along with high-resolution seismic attribute volumes.

## Materials and methods

### Site description

**Fig. 1:**
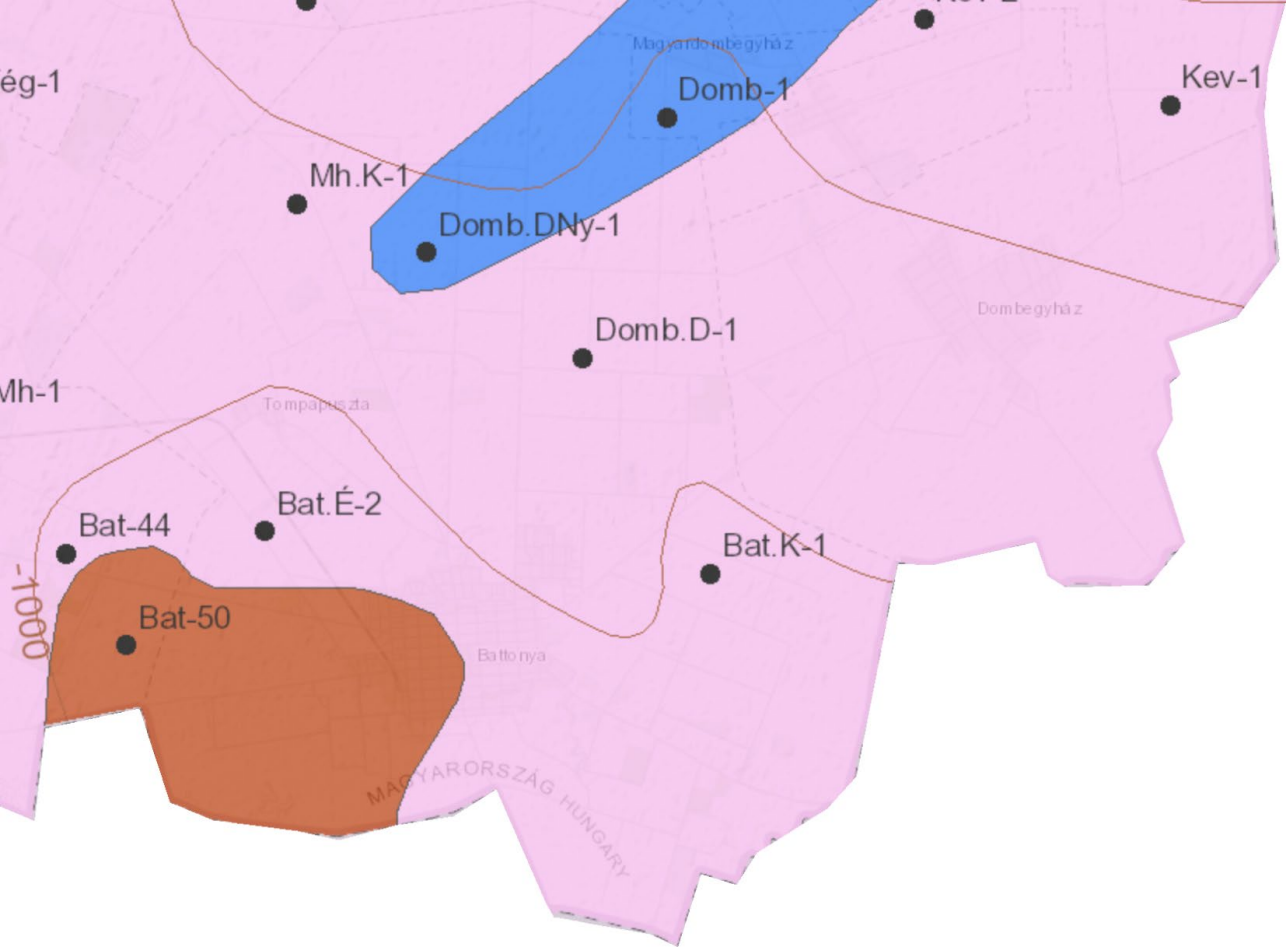
Geographical location of the investigated region and geological map of the Pre-Tertiary basement edited based on previous map (Haas et al. 2014).

The study area is located between longitude 21.22 E and latitude 46.3 N on the southeastern part of Hungary in the Pannonian Basin which is a young sedimentary basin formed since the Mid Miocene to Quaternary. Specifically, on the Battonya-Pusztaföldvár Ridge between the Makó and Békés Basins (Fig. 1).

## Geological overview

Hungary is located in the Pannonian Basin, which is an intramountain basin that is still in its infancy bounded by the Alps, Carpathians, and Dinarides^30^. The basin’s basement is made up of Pre-Cenozoic rock blocks that are only visible on the surface in the Transdanubian Range, Sopron– Kőszeg, Bükk, Aggtelek–Rudabánya, Mecsek, and Villány mountain ranges. Plate tectonic movements resulted in lithosphere shrinkage and intense subsiding, resulting in the formation of the Pannonian Basin over the last 19 million years^30^. In the lowlands, newer Cenozoic strata cover thousands of meters of the Pannonian Basin. The basement is fairly complex beneath the massive and reasonably homogeneous Late Cenozoic layer. It depicts a mosaic pattern composed of heterogeneous structural features, a mix of allochthonous terranes obtained from various regions of the Tethyan realm^30^. The Tethys Ocean lies between two massive tectonic plates (African and Eurasian). The Alpine Tethys began to close in the Early Cretaceous. The pre-Cenozoic basement of Hungary is made up of three mega-units; (1) the Tisza Mega-unit, which is derived from the European Plate; (2) the Alcapa Mega-unit, which is derived from the African Plate; and (3) the Mid-Hungarian Megaunit, which is derived from the shear zone between these two^30^.

The subsurface Battonya–Pusztaföldvár basement ridge is composed of nappe structures, where a northern, northwest vergence overthrusted the metamorphic basement complex’s material to the Palaeo-Mesozoic sequence. The region is a part of the Békés–Codru Unit nappe-imbricated structure, which is part of the Tisza Mega-unit (Fig. 1). The compressional tectonics of the Cretaceous period are primarily responsible for the formation of the nappe system. Tectonic structures-oriented NE–SW are found within the nappe structure, and Cenozoic transverse faults-oriented NW–SE are also detectable^31^. The crystalline rocks in the study area are classified under the Battonya unit, which falls within the Békési Terrane. The granitoid rocks have an origin that is a combination of the Earth’s crust and mantle^32,33^. They are situated along the central axis of the Battonya- Pusztaföldvár high and are surrounded by a broad zone of migmatite and crystalline schist on the northeast and southwest sides^30,34^. Generally, above the basement uplifts, including the Battonya-Pusztaföldvár ridge, in the innermost part of the basin, a starving basin developed with condensed sedimentary sequences (limestone, marl, clay marl). In these basins, these formations constitute the Endrőd Marl Formation, forming the Pannonian base. This formation is formed under extremely variable water depth conditions (15–800 m), with a thickness of 20–100 m on the ridge but reaching up to 700 m towards the basins. Its sequence typically begins with sporadic deposition of limestone and marl layers, each about 40–50 m thick (Tótkomlós Member), gradually transitioning upwards into deep-water (hemipelagic) clay marl (Nagykörü Member). The Endrőd Marl Formation is present in almost all wells proposed for concession and is the main seal for the geothermal system in the study area^30,31,35^.

## Hydrological settings

The Pannonian basin is dominated by two distinct fluid flow regimes. The first is an upper flow system that is driven by gravity. The second is a deeper system that is driven by pressure, specifically overpressure. This deeper system is found in the fine grain deep-sea deposits and deeper formations^29^. It is likely that the elevated overpressure (approximately 10 MP ) is a result of formations compression due to active tectonics^36^. The Pannonian basin has porous formations that hold water at temperatures ranging from 130 to 140 ºC, because of the high geothermal gradient. In certain cases, deep aquifers that are made up of karstified and fractured carbonated and metamorphic basement can even reach temperatures as high as 300 ºC. These conditions create an ideal environment for the development of medium- and high-energy geothermal systems^29^. In a study conducted by^29^, the geothermal resources of Hungary were assessed. The researchers determined the total heat content in the rock matrix and water within geological formations of different ages, up to a depth of 5 km. It was shown that the amount of stored heat increases as the depth increases. The measured quantity of stored heat was determined to be around 100,000 exajoules. The majority of the heat is contained within the rock matrix, while a small portion, approximately 5%, is stored in the pore waters^37^.

## Three-dimensional seismic model

The 3D Seismic and well data play a crucial role in assessing the adopted three geothermal risk sources parameters: heat source, reservoir fracture permeability, and seal in the study area. Figure 1S shows an example of the seismic to well tie result. The tie shows a high cross correlation of 83% between the seismic data and the synthetic seismogram. This study utilizes a high-resolution 3D post-stack seismic dataset acquired in 2006 and processed in 2007. This data offers detailed insights into the subsurface geology of the study area.

The seismic data interpretation process involved two key phases:

(i) **Conventional Interpretation**: this initial phase focuses on identifying major faults and the top boundaries of the seal and pre-Tertiary basement.
(ii) **Attribute Generation**: this advanced phase involved applying various seismic attribute techniques such as frequency filtering attributes, structural attributes, and colored inversion.

A resource attribute table is generated to provide a concise overview of key characteristics such as heat, permeability, and seal, along with the corresponding data requirements for their identification. These data requirements include parameters such as heat flow, reservoir temperature, fault information, and seal distribution, among others (Table 1). The critical element risk maps are derived from the resource attribute worksheet, which outlines the key components of the heat source, fracture reservoir permeability, and seal. Additionally, our assessment of the anticipated uncertainty associated with each data type contributes to the formulation of these risk maps.

**Table 1:** A resource attribute table to summarize important properties and the data needed to identify them.

Resource attribute	Technique	Data
Heat source	- Heat flow - reservoir temperature	- Heat flow density map - geothermal gradient - well data
Reservoir fracture permeability	- Coherence attribute - Ant tracking attribute - Colored inversion	- Reflection seismic - Regional mapping - stress maps
Cap rocks/seal	- Seal spatial distribution - Seal thickness	- Reflection seismic - well data

It is necessary to establish a consistent set of terms when referring to the different GIS layers as data are processed in the creation of the CRS maps. The following terms are applicable to all data types and facilitate communication among different domain experts. In this study, we utilized the terminology established by^28^. The layers are defined as follows: (1) raw data layers, (2) evidence layers, (3) CRS maps, and (4) CCRS map.

**Fig. 2:**
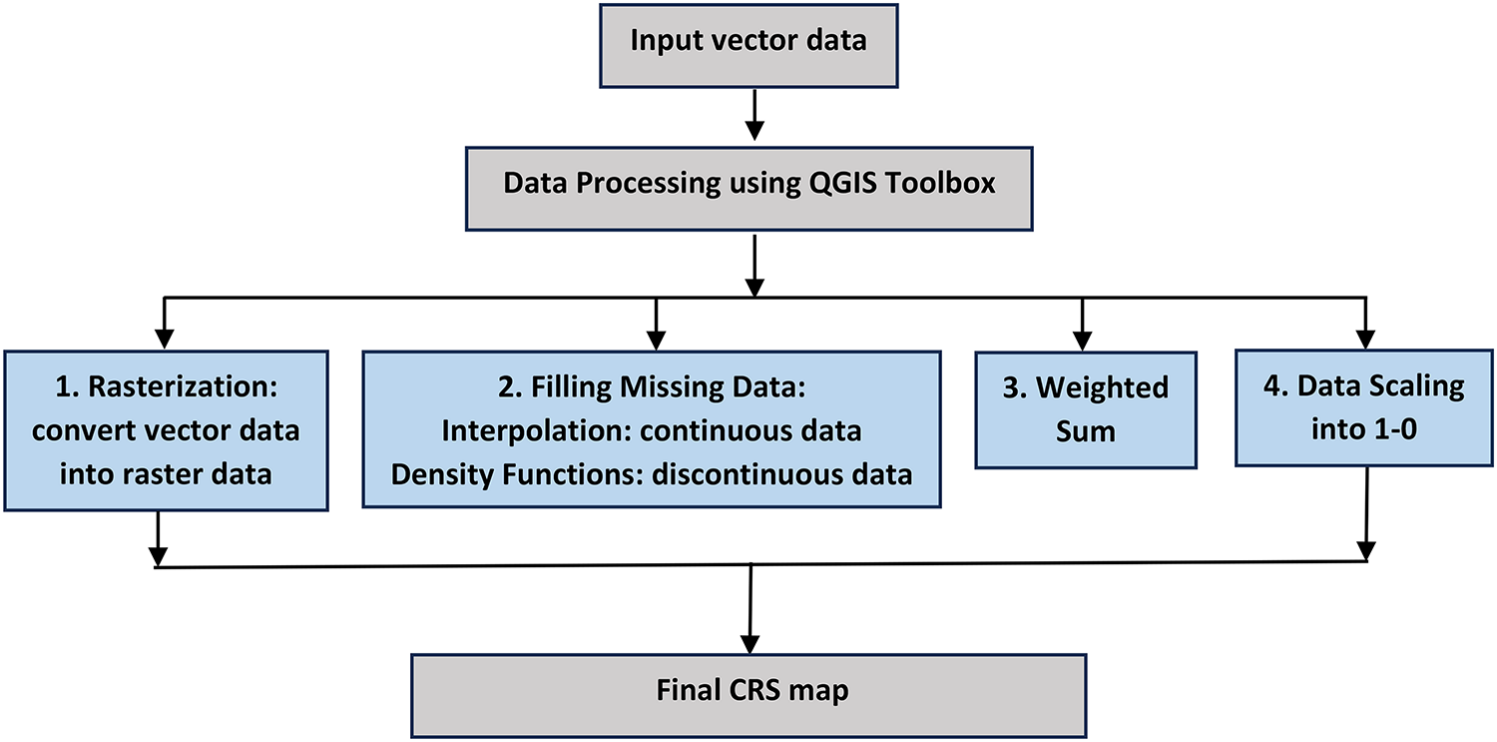
Basic diagram illustrates the processing steps for data transformation to create the CRS maps of each risk component.

The primary interpretation software utilized in our workflow is Petrel (Schlumberger 2016) and QGIS 3.34.8^38^ alongside with 3DHIP calculator. Petrel software is employed for both stratigraphic and structure interpretation, enabling the generation of initial raw data layers. QGIS, also known as Quantum Geographic Information System, is a freely available open-source software that enables users to perform various tasks related to geospatial information. These tasks include creating, editing, visualizing, analyzing, and publishing geospatial data. QGIS is utilized to merge data layers obtained from petrel software and other publicly available published data sources. The purpose of this merging process is to generate a set of evidence layers that can be further analyzed at later stages. Various processing techniques are employed to handle different types of data (Fig. 2).

**Fig. 3:**
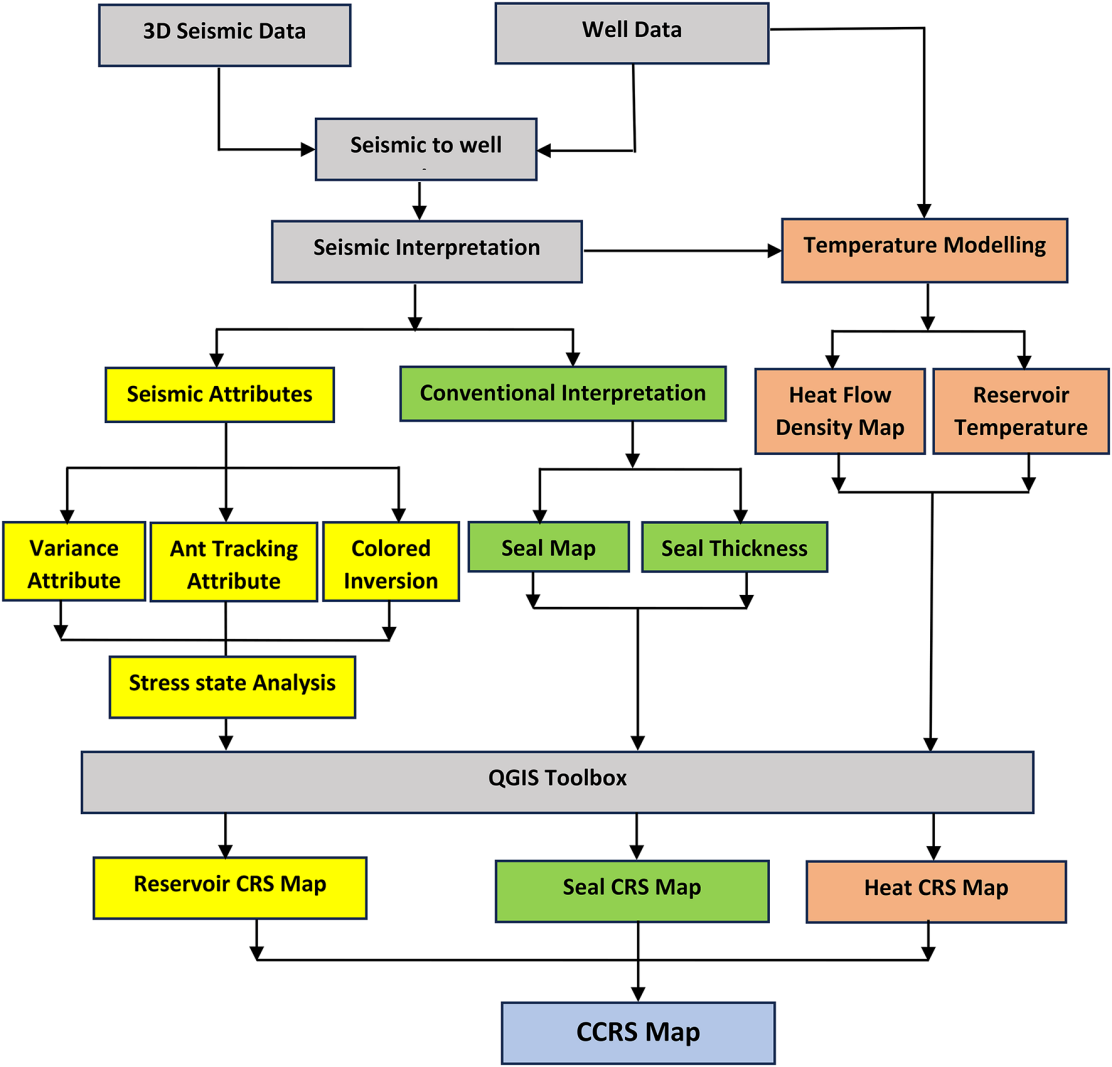
The detailed workflow for evaluating the geothermal potential and creation of the risk maps.

The process of converting data layers into evidence layers can be accomplished using density functions, which determine the distribution of a certain feature (such as fault segments), or through interpolation, which calculates a continuous output based on point data inputs (such as heat flow or reservoir temperatures). Density functions are employed for datasets that exhibit discontinuity and where the spatial distribution of the data holds significance. Interpolation is employed to estimate values between data points that are inherently continuous but can only be obtained at discrete intervals^25^. To generate a CRS map, the evidence maps of the various elements are assigned weights in proportion to their respective influences, and then these weighted maps are merged. The normalized CRS for each risk component (heat source, reservoir fracture permeability, and seal) will be assigned weights and then combined to calculate the final CCRS. The CCRS will be further normalized to provide the final favorability geothermal map of the research area. The CCRS map delineates multiple high-priority objectives for a forthcoming, concentrated investigation. Figure 3 displays the comprehensive sequence of steps for this technique in the form of a flowchart. All weighting coefficients were utilized following a trial-and-error process to confirm that the coefficients yielded reasonable results based on expert experience, demonstrating that known, deep, high-temperature locations exhibit above-average favorability. Table 2 presents the weighting coefficients employed in weighted sum computations.

**Table 2:** Weighting coefficients used in weighted sum calculations. weight coefficient values relate to the features in the data column in the order they appear.

Data	W	W	W
CRS heat source: Heat flow, reservoir temperature	2	3	
CRS seal: Spatial distribution, vertical Thickness	1	1	
CRS reservoir fracture permeability: Fault probability, Ant tracking, Colored Inversion	2	4	1
CCRS: Heat source, seal, reservoir fracture permeability	3	1	5

In this study, the 3DHIP-Calculator^39^, which is an open-source tool is used to estimate the geothermal potential of the study area in terms of thermal energy stored within the reservoir in Petajoules per square kilometer (PJ/km^2^), and, more importantly, the recoverable energy potential in megawatt thermal per square kilometer (MWth/km^2^). It utilizes the volumetric Heat-In-Place (HIP) method, which was originally developed by the United States Geological Survey (USGS) in 1978. The software also incorporates a Monte Carlo simulation approach introduced by Nathenson in the same year^41^. To perform the calculations, 3D geological and 3D thermal models of the study area in a voxel format are used as inputs. This comprehensive approach considers the complete geological heterogeneity of the reservoir to accurately estimate its geothermal potential. The 3DHIP-Calculator is used by many authors for geothermal potential calculation like^39,42–44^.

The volumetric Heat-In-Place (HIP) method quantifies the thermal energy contained in both the rock and water volumes, and subsequently determines the amount of this energy that can be extracted. The thermal energy in a given location can be calculated using the equation (Eq. 1) proposed by^40^. This equation can be solved either deterministically or by employing a probabilistic approach in conjunction with Monte Carlo simulations.

1 $$\:\text{H}\text{I}\text{P}=\text{V}\cdot\:\left[{\varnothing}\cdot\:{{\uprho\:}}_{\text{F}}\cdot\:{\text{C}}_{\text{F}}+(1-{\varnothing})\cdot\:{{\uprho\:}}_{\text{R}}\cdot\:{\text{C}}_{\text{R}}\right]\cdot\:(\text{T}\text{r}-\text{T}\text{i})$$

Where HIP is the heat in place or stored energy (PJ), V is the cell or voxel volume (m3), ∅ is the porosity (fraction), is the fluid density (kg/m3), is the fluid specific heat capacity (kJ/kg·ºC), Tr is the cell or voxel temperature (ºC), is the rock density (kg/m3), is the rock specific heat capacity (kJ/kg·ºC), is the temperature of the cell or voxel in the 3D thermal model (ºC), and Ti is the reinjection temperature (ºC).

Then, the estimated HIP value is used to determine the recoverable heat (Hrec) using the following Eq. (2)^45,46,43^.

2 $$\:\text{Hrec}=\frac{\text{H}\rm{I}\text{P}\cdot\:{\text{C}}_{\rm{e}}\cdot\text{R}}{\text{Tlive}\cdot\:\text{P}\text{f}}$$

Where $\:{\text{C}}_{\text{e}}$ is the conversion efficiency (fraction), R is the recovery factor (fraction), Tlive is the mean plant lifetime or total project live (years), and Pf is the plant factor (fraction).

## Heat source

A proximal heat source is the principal requirement for a high temperature geothermal system that is within economically accessible drilling depths. In the Battonya High area, a significant amount of heat can be derived from the upward fluid flow through the faulted zone of the crystalline blocks by convective heat transport. The Pannonian Basin is widely recognized for its considerable geothermal potential^12,46^ owing to its advantageous thermal water resources and favorable geological conditions. The Pannonian Basin exhibits an elevated geothermal anomaly, as evidenced by the mean heat flow density of 90–100 mW/m^2^, which ranges from 50 to 130 mW/m^2^. The earth’s continental crust has a relatively thin composition, measuring 22–26 km in thickness^46^. Additionally, it is overlaid by formations with low thermal conductivity. The prevailing conditions resulted in an unusual geothermal gradient anomaly of around 50 °C /km^29,49^. The study area is a hybrid of the structurally controlled Pannonian Basin geothermal system, and basement driven systems. Thermal energy is provided by a granitic high temperature pre-Tertiary basement. Heat flux is preserved by The Endrőd Marl Formation, a seal that both insulates the reservoirs and prevents venting to the surface. The heat flow density values reached up to 105 mW/m^2^ within the study area. Alongside the carefully measured high top basement temperature of up to 150 °C, this provides strong evidence of a robust and long-lasting heat source for geothermal systems in the study area.

**Fig. 4:**
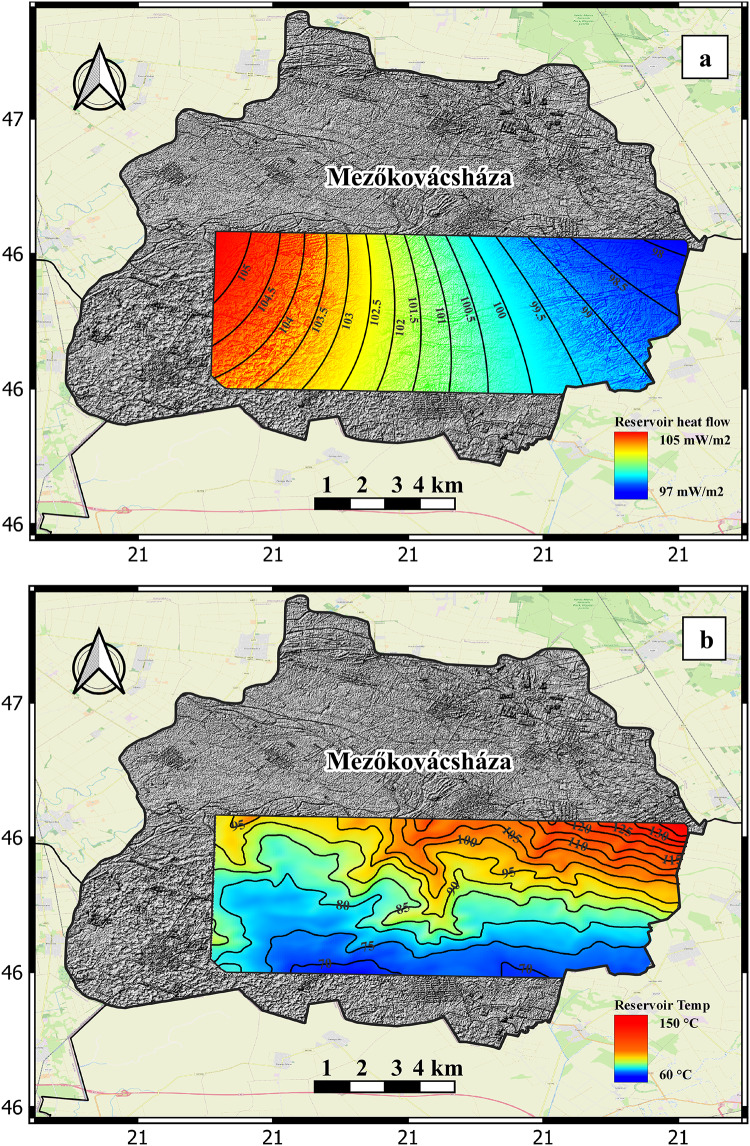
(a) Heat flow distribution in the study area in mW/m^2^. (b) Temperature contour map of the basement calculated from geothermal gradient value of the study area in °C.

The data collected within the depth range of 1–2 km near the basement surface reveals a geothermal gradient of 55–70 °C/km, which decreases as the depth increases. The production testing of well Mbh.K/1, located at the edge of the study area, measured a temperature of 140 °C at a depth of 2120 m. This indicates a geothermal gradient of 60 °C/km at a depth of 2000 m^48^. The temperature of the Mezőkovácsháza top basement is calculated using the geothermal gradient measured from the available wells then multiplied by the depth converted top basement to create the temperature map of the basement. The CRS map for heat source is created by combining data representing the heat flow (Fig. 4a) and the calculated reservoir temperatures (Fig. 4b) of the study area.

**Fig. 5:**
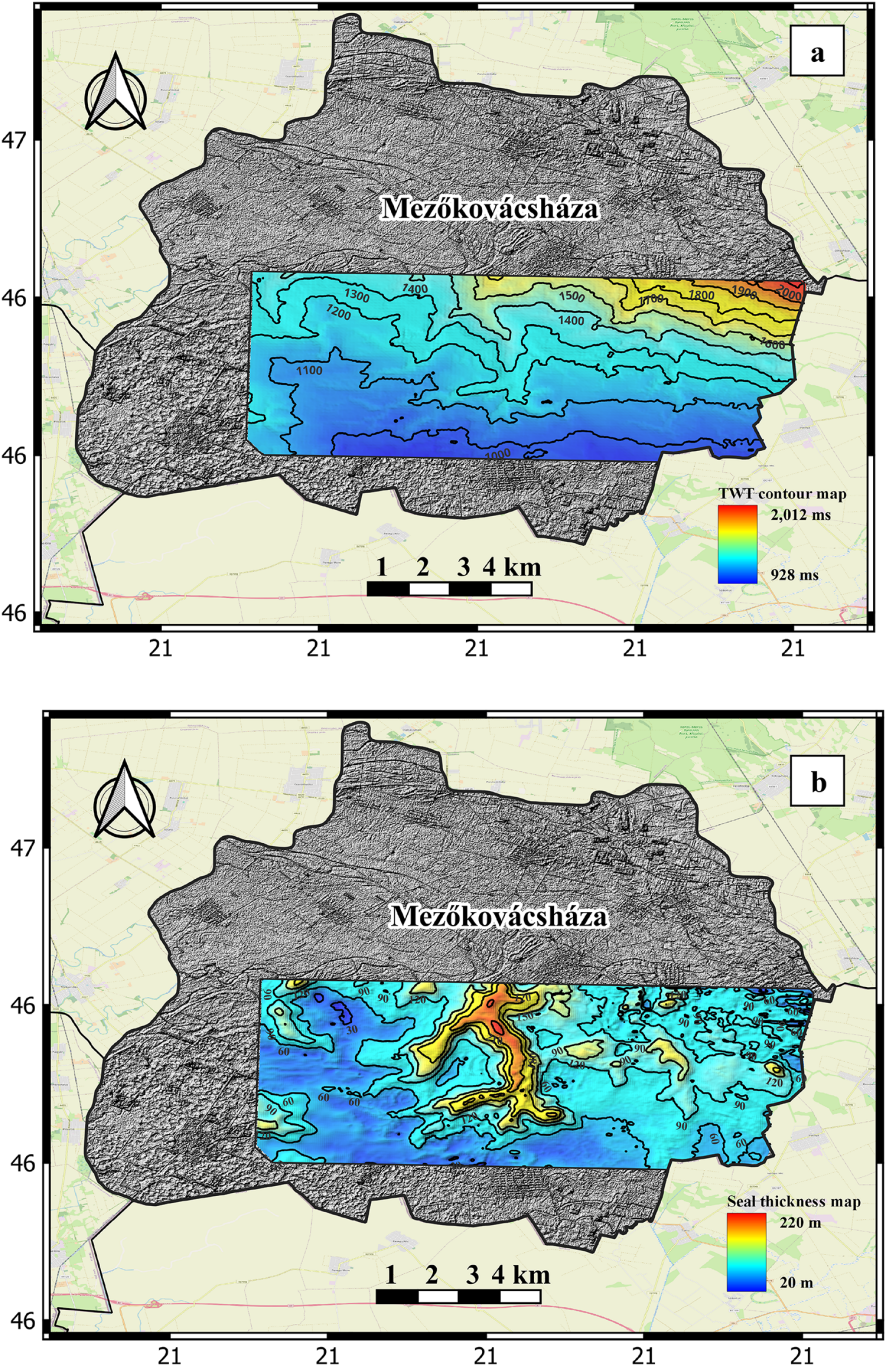
(a) Two-way time structure contour map in ms showing the seal distribution in the study area and (b) thickness map in meters of the seal horizon. Created by QGIS 3.34.8 (https://www.qgis.org/)^38^.

Figure 4 seems to present a contradiction between heat flow distribution and temperature results, where 4a shows higher heat distribution at the NW end, while 4b indicates higher temperatures at the NE end. However, this apparent inconsistency can be explained by considering the overall heat flow and depth variations within the study area. The heat flow distribution, as shown, is relatively homogeneous, ranging from 97 to 105 mW/m² across the region. The higher temperature values observed in the NE are primarily attributed to the increasing depth of the geothermal reservoir towards the NW, as illustrated in Fig. 5b. This suggests that the temperature is more influenced by the geothermal gradient, which increases with depth, rather than by variations in heat flow alone. Therefore, the higher temperatures in the NE are a result of greater depths, with heat flow playing a secondary role in determining the reservoir temperature.

### Seal

For a working and preserved geothermal system, an effective intact seal is a of great importance^49^. If the cap rock of a geothermal system is absent or fractured, the hot fluids will escape to the surface through hot springs or mix with cold waters in the near surface shallower aquifers. Lower thermal conductivities than the volcanic reservoir rocks of overlying sediments, also act as a thermal blanket to retain heat and preserve the geothermal system. The Endrőd Marl Formation is divided into two members Nagykörü argillaceous Marl and Tótkomlós Calcareous Marl and present in all wells in the study area. The Endrőd Marl Formation serve as effective reservoir seals in the study area^31^. The distribution of Endrőd Marl Formation in the study area is documented by well logs interpretation and detailed studies of core description reports. Figure 5a shows the two-way time structure contour map and Fig. 5b thickness of the seal horizon depicting its spatial distribution and capacity acting as a thermal blanket for the underlaying hot basement. The generated seal two-way time structure contour and thickness of the seal horizon maps are used to create the CRS map for the seal component.

## Reservoir fracture permeability

Fractures, both natural and artificially created, serve as the primary pathways for fluid flow in geothermal reservoirs. Geothermal reservoirs are almost exclusively reliant on fracture permeability, associated with fracturing related to tectonic and magmatic processes. Fractures are difficult to characterize in the subsurface, but their presence can be mapped using seismic data through structural interpretation. The fracture systems typically exhibit anisotropic behavior, which is crucial for the development of geothermal reservoirs. The main goal of geothermal producers is to effectively locate geothermal wells in productive and high-temperature areas known as sweet spots^50^. Analysis of fault trace maps and quantitative structure/stress analysis have been used to help locate permeability associated with large, mapped structures. permeability is typically highest within step-overs (transfer zones), accommodation zones, and fault intersections^51^; these are high priority targets for identification and mapping. Due to different tectonic phases in the past, especially Cretaceous thrust sheet movements, the pre-Tertiary basement is fractured with hardly predictable features. We choose the top 500 m thickness of the basement interval as our reservoir to conduct our research and calculate this interval potential for geothermal exploration in the whole study area. The CRS map for reservoir fracture permeability is created by combining data representing mapped faults and structures inferred from seismic data interpretation.

The fractures within the reservoir are of utmost importance in determining the efficiency of a geothermal system, as they contribute to the permeability risk factor. However, accurately measuring these fractures can be quite challenging. Additionally, they pose the greatest risk in the field of geothermal exploration. In order to tackle this issue, a range of seismic attributes are produced with the purpose of mapping the fracture zones within the chosen basement reservoir.

## Structural attributes for fault detection

The coherence attribute is used to measure the similarity of adjacent waveforms or traces over specified lateral and/or vertical windows. Thus, it can detect discontinuity in seismic data associated with faulting or stratigraphy. The coherence attribute has been shown to be useful in visualizing channels and faults^52–54^. It can also be used to directly display major fault zones, fractures, unconformities, and major sequence boundaries^55^. Calculations related to semblance are crucial for the accuracy of (τ, p) analysis algorithms^56,57^ and f -x deconvolution algorithms. We start by defining an analysis window that contains a specific number of traces centered around the analysis point. When centering the local (x, y) axis around this analysis point, the semblance, σ (τ, p, q), is defined as follows:

3 $$\:\sigma\:(\tau\:,p,q)=\frac{{\left[\sum\:_{j=1}^{J}\:u\left(\tau\:-p{x}_{j}-q{y}_{j},{x}_{j},{y}_{j}\right)\right]}^{2}+{\left[\sum\:_{j=1}^{J}\:{u}^{H}\left(\tau\:-p{x}_{j}-q{y}_{j},{x}_{i},{y}_{j}\right)\right]}^{2}}{J\sum\:_{j=1}^{J}\:\left\{{\left[u\left(\tau\:-p{x}_{j}-q{y}_{j},{x}_{j},{y}_{j}\right)\right]}^{2}+{\left[{u}^{H}\left(\tau\:-p{x}_{j}-q{y}_{j},{x}_{j},{y}_{j}\right)\right]}^{2}\right\}},$$

**Fig. 6:**
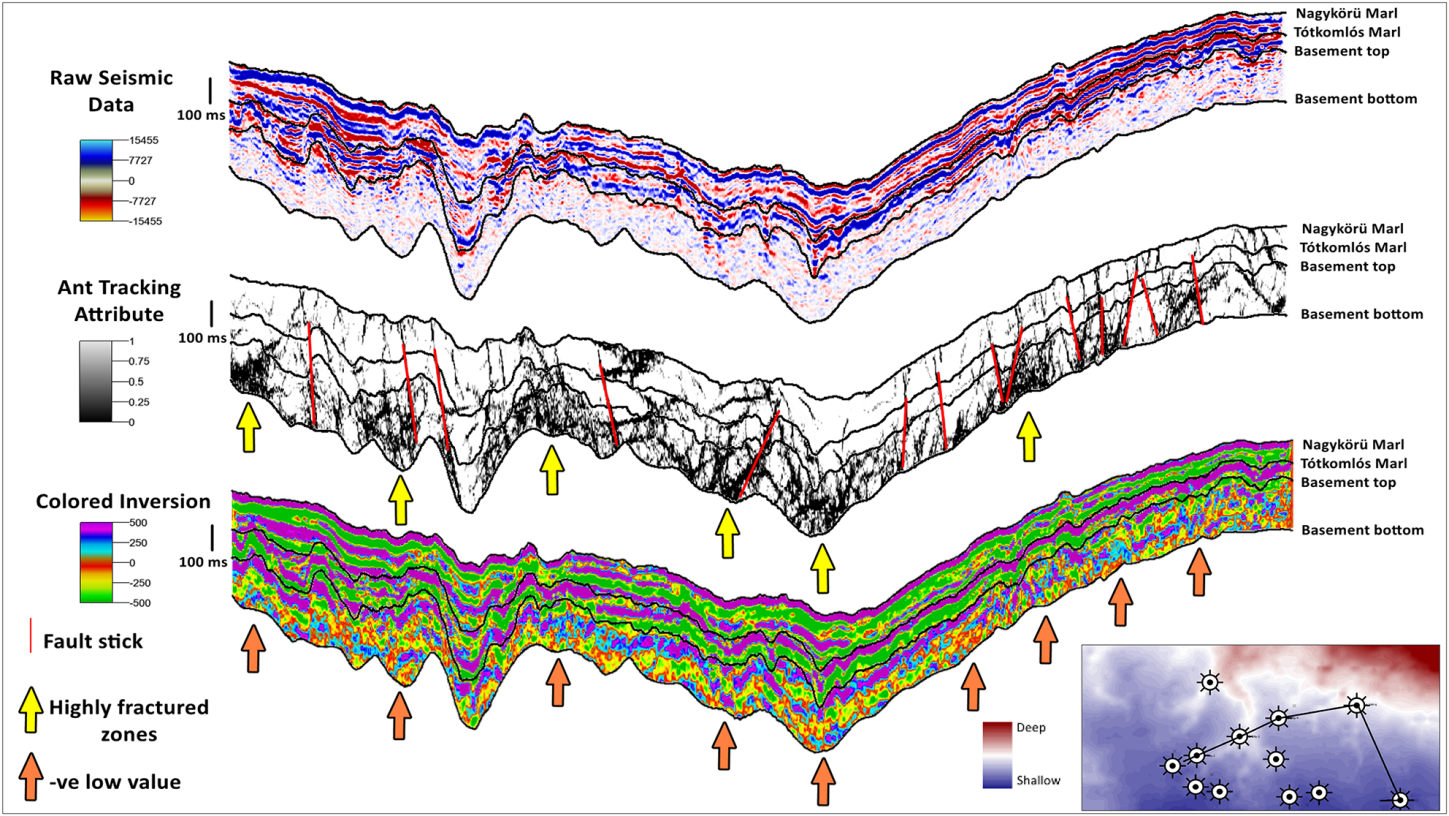
The CI attribute result compared to the ant tracking attribute result and seismic raw data through an arbitrary line cutting through the study area. the arbitrary line configuration and wells distribution are depicted in the lower right corner map.

Where, the triple (τ, p, q) represents a local planar event at a specific time τ. The values p and q indicate the apparent dips in the x and y directions, measured in milliseconds per meter. The superscript H refers to the Hilbert transform or quadrature component of the real seismic trace, u. By calculating the semblance of the analytic trace, we can obtain reliable estimates of coherency, even when it comes to the zero crossings of seismic reflection events. Coherence attribute in this study is used to highlight structural features like faults and fractures. The coherence attribute is then used as an input to the ant tracking attribute to create a fracture cube that specifically identifies potential fracture zones within the basement. The ant-tracking attribute is developed by Schlumberger Stavanger Research and is commercially available in the Petrel™ software is a highly advanced attribute that utilizes the swarm intelligence algorithm to improve discontinuities^58^. The mission of the ant-trackers, or artificial ants, is to locate and enhance the discontinuity traces within the 3D coherence attribute volume. With this approach, we establish azimuthal parameters to guide the movement of the ant-trackers within the 3D coherence volume. This allows the artificial ants to effectively search for discontinuities in the edge-detection volume. Figure 6 illustrates the ant tracking attribute and the picked faults and fractures interpretation in comparison to the seismic raw data. The comparison is made along an arbitrary line that cuts through the area of interest. The ant tracking attribute is capable of accurately detecting faults and fractures in the target reservoir that are not visible in the raw seismic data. It also identifies highly fractured zones in the basement target (yellow arrows), which are a result of various tectonic phases in the past, particularly Cretaceous thrust sheet movements. The red fault sticks represent interpreted faults and fractures revealed after ant tracking attribute and yellow arrows indicate highly fractured zones within the target basement reservoir. The utilization of the ant tracking attribute enables the interpretation of faults and fractures, providing increased confidence in assessing the risk component of fracture reservoir permeability.

### Colored Inversion (CI) proxy for fractures determination

Seismic inversion is a process that transforms reflectivity data into physical properties of the earth, specifically acoustic impedance (AI), which is the result of combining seismic velocity and bulk density. It is important to consider that reflectivity provides information about boundaries, while impedance can be converted to earth properties such as porosity and fluid content using established petrophysical relationships^59^,^60^. published a method for band-limited inversion of seismic data called colored inversion (CI). The authors demonstrated that the widely used sparse-spike inversion process can be simplified by deriving a single operator from well logs. This operator allows for the calculation of relative acoustic impedance through a straightforward convolution with the reflectivity data. Wavelet extraction or a low frequency model is unnecessary in this case, and the output remains unbiased by the trend in the well data. Colored inversion is used in this study as well data is scarce, and it is strongly advisable technique in areas where there is insufficient well control to produce a dependable absolute inversion^61^. The result of the colored inversion is used as an indication for fractured zones in this study as where the basement is less acoustic impedance value mean more fractured zones than the non-fractured zones which will have relatively higher impedance values. Figure 6 depicts the colored inversion attribute results compared to seismic raw data and ant tracking attribute through an arbitrary line cutting through the area of interest. The colored inversion attribute shows low negative values in the fractured basement target due to low acoustic impedance values of the fractured basement (orange arrows). The colored inversion attribute results show a good matching with the faulted and highly fractured zones interpreted from ant attribute interpretation. In the current study, the colored inversion scale in Fig. 6 shows that the range of relative acoustic impedance is from − 500 to 500, where the positive values indicate the absence of basement fractures of low to no porosity which is from 0 to 500. In contrast, the colored inversion result shows low negative values reach − 500 which indicate fractured basement. So, the results of this attribute confirm the ant track attribute findings to some extent and can be used as a fracture presence proxy.

### Stress state interpretation

**Fig. 7:**
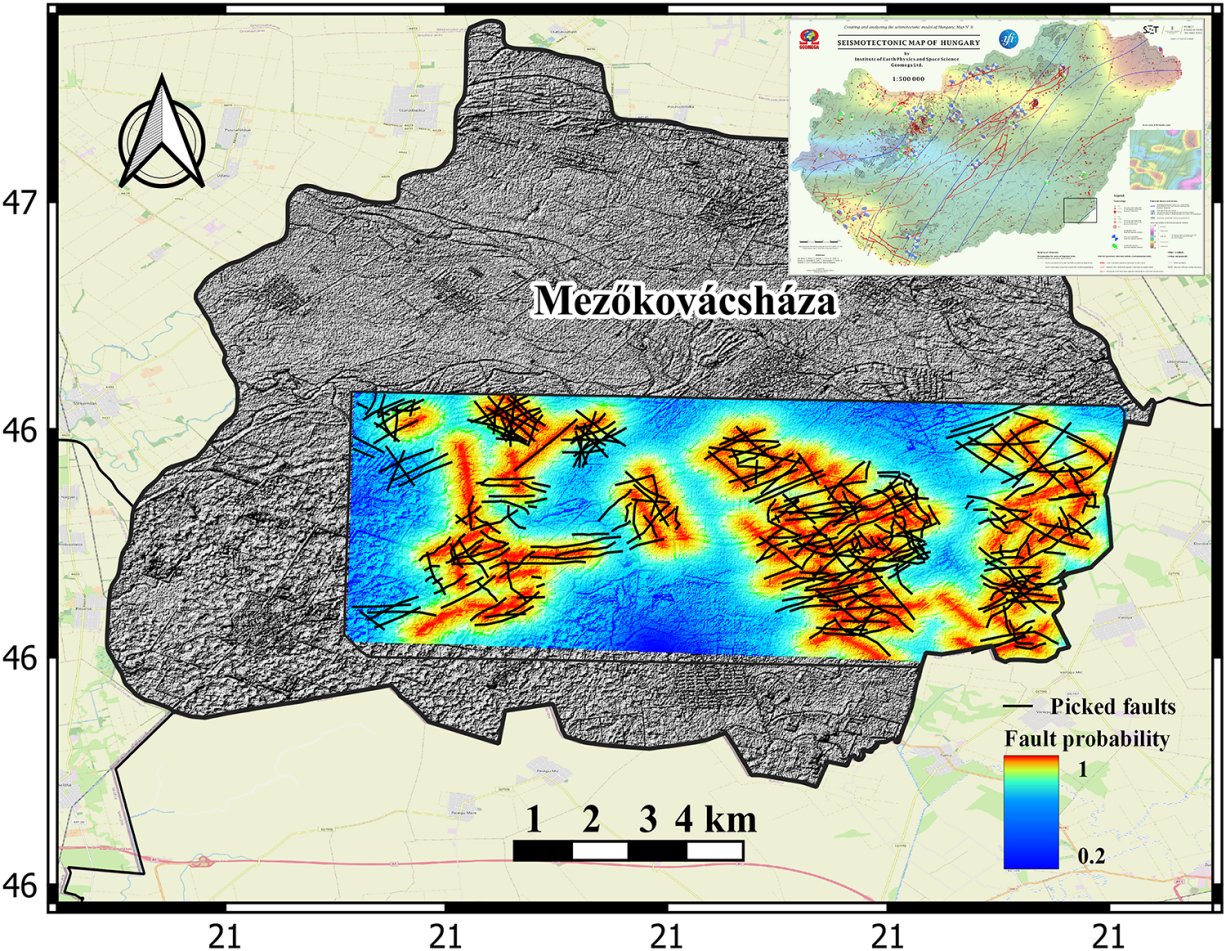
fault and fracture probability map based on stress state interpretation and fault density distribution showing the most likely open fractures. The seismotectonic map of Hungary is shown at upper right corner. Created by QGIS 3.34.8 (https://www.qgis.org/)^38^ .

The utilization of fault trace maps and quantitative structure/stress analysis has aided in the identification of permeability linked to significant, mapped structures. The surface faults and structural lineaments are assessed for the tendency to experience slip and dilation within the regional stress field. These tendency values are then utilized as weights in the final CRS map for the reservoir fracture permeability risk source parameter. Figure 7illustrates the seismotectonic characteristics of Hungary^62^ (upper right corner), where the prevailing stress regime is compression, with the maximum horizontal stress direction (SH_max_) oriented in the NE-SW direction. Fluids typically exhibit a preference for flowing in the direction of the maximum horizontal stress (SH_max_), where the vertical stress is at its lowest and the resistance to flow is minimized^63^. Under compressional stress conditions, open faults and fractures develop at right angles to the minimum stress component, which in this case is the vertical stress. These faults and fractures are more prone to slipping and expanding^64,65^. Therefore, we assigned more weight to the faults and fractures that align with the SH_max_ direction and are perpendicular to the vertical stress in the study area, compared to those in other directions (Fig. 7). The structural seismic attributes and stress state interpretation are utilized to generate fault and fracture maps, which are then combined to produce the CRS map of the reservoir fracture permeability component.

## Results

In this study, the PFA approach is performed to construct a geothermal play fairway model for the Mezőkovácsháza region. The purpose was to identify the most favorable prospects and mitigate the risks and expenses associated with geothermal development. This study aimed to discover three essential resource components in the study area as the main risk components for geothermal exploration and development in this play type: cap or seal, reservoir fracture permeability, and heat source. CRS maps for each component are generated by aggregating data from all the constituent items that contribute to that particular component. The CRS maps of the three risk components in the study area exhibit varying degrees of risk and favorability. The weighted CRS maps are summed to produce the final CCRS favorability map for geothermal exploration in the study area.

### Heat source

The CRS map for the heat source is generated by merging data that represents the heat flow in the study area with the estimated temperature of the reservoir (Fig. 4). The heat flow distribution map displays a range of high heat flow values, ranging from 90 to 105 mW/m^2^. These values exhibit a gradual increase from west to east across the study area. The estimated reservoir temperature in the study area is high and exhibits a continuous increase with depth, as determined by the measured geothermal gradient.

**Fig. 8:**
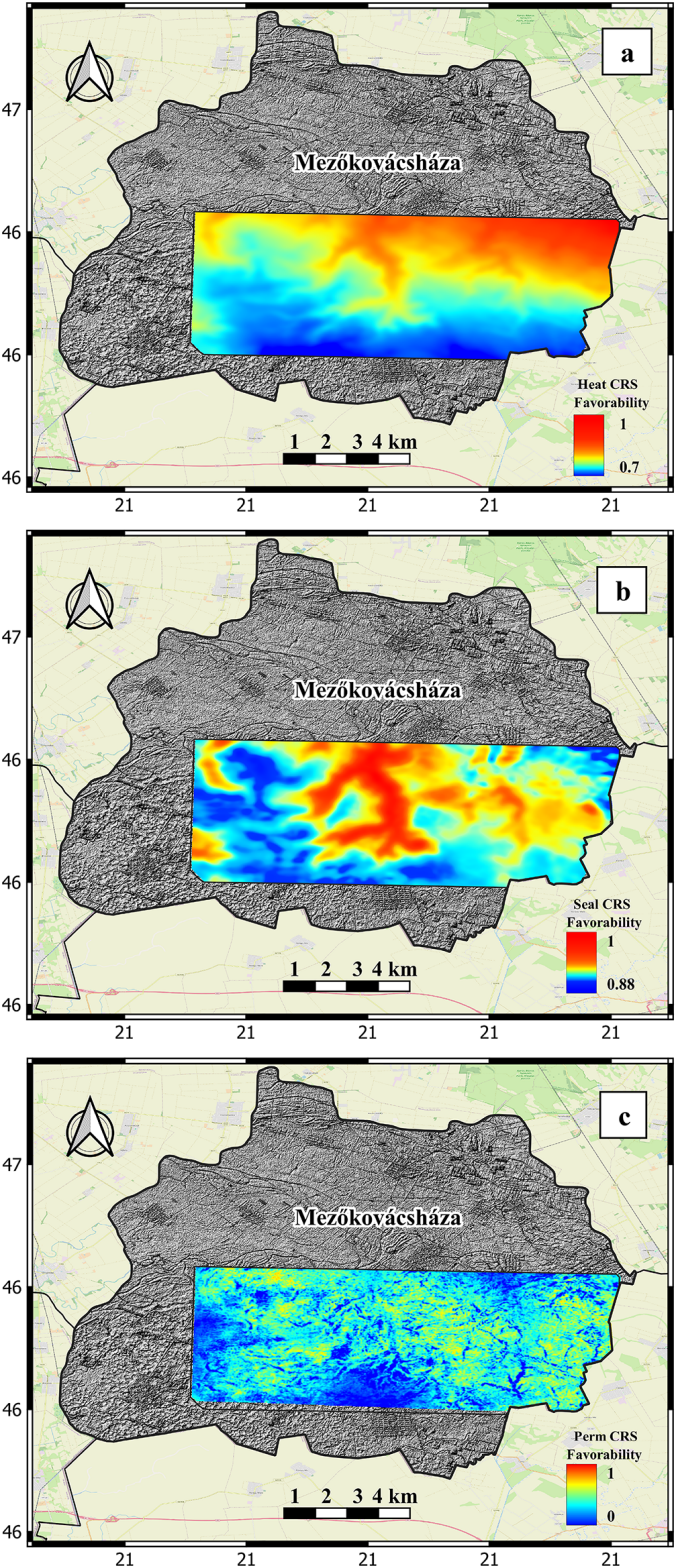
(a) The heat source CRS map component. (b) the seal CRS map component. (c) the reservoir fracture permeability CRS map component.

The CRS map of the heat source component, as shown in Fig. 8a, exhibits high favorability values. These values range from 0.7 at shallower depths to 1 at deeper parts of the investigated target. Based on the analysis of the heat source CRS map, it can be determined that the heat source in the study area shows promise and does not pose a significant risk for geothermal exploration and development.

### Seal component

The CRS map for seal is generated by integrating data that represents the distribution and thickness of impermeable upper basement marl deposits. The distribution of the Endrőd Marl Formation in the study area has been documented through the interpretation of well logs and detailed studies of core description reports. The two-way time structure contour map illustrates the spatial distribution of the seal layer, revealing a full seal coverage without any eroded portions (Fig. 5a). Figure 5b displays the thickness distribution of the seal horizon, which demonstrates its ability to function as a thermal blanket for the underlying hot basement.

The (CRS) map of the seal component, depicted in Fig. 8b, demonstrates elevated favorability values. The values observed in various parts of the investigated target range from 0.88 to 1. A value of 0.88 indicates the parts that has low vertical thickness and are partially prone to be fractured based on the structural attribute analysis, while a value of 1 indicates the parts that are of high vertical thickness and has almost no fractures in the study area. The analysis of the seal CRS map indicates that the seal source in the study area exhibits favorable characteristics and is not considered a notable risk for geothermal exploration and development similar to the heat source component.

### Reservoir fracture permeability

The creation of the CRS map for reservoir fracture permeability involves the integration of various data sources. These sources include mapped faults, stress orientation and magnitude analysis, fracture cube analysis, and colored inversion results. The ant tracking attribute demonstrates the ability to precisely identify faults and fractures within the target reservoir that may not be discernible in the original seismic data. It additionally detects and identifies zones of high fracture density within the basement target. These fractures are a consequence of different tectonic phases that occurred in the past, specifically the movements of Cretaceous thrust sheets. The utilization of the ant tracking attribute facilitates the interpretation of faults and fractures. The colored inversion attribute is utilized to visualize low negative values in the fractured basement target. These low values are a result of the fractured basement having low acoustic impedance values. The technique of colored inversion is employed in this study due to the limited availability of data. It is highly recommended for use in areas where there is insufficient well control to generate a reliable absolute inversion. The colored inversion result is utilized in this study to identify fractured zones. In this context, areas with lower acoustic impedance values indicate a higher likelihood of fractured zones, while non-fractured zones tend to exhibit relatively higher impedance values.

The CRS map of the reservoir fracture permeability component, as shown in Fig. 8c, exhibits a dispersed distribution. The distribution of values in this CRS map demonstrates the existence of regions with high favorability values, as well as regions with values close to zero. The values in the range from 0 to 1 represent the extent of fractured areas within the study area. A value of 0 indicates the complete absence of any fractured areas, while a value of 1 indicates the presence of highly fractured areas in specific sections of the study area. The analysis of the CRS map of reservoir fracture permeability indicates that the reservoir fracture permeability in the study area is a significant risk factor for geothermal exploration and development. The reservoir fracture permeability, in contrast to the seal and heat source components, does not have a uniform distribution and shows considerable variation. Ensuring precise measurement and proper handling of this component is crucial in order to effectively minimize any potential risks. The CRS map for reservoir fracture permeability is given more weight compared to the heat source and seal CRS maps due to its significant importance and its role in identifying favorable regions. This map is then combined with the heat source and seal CRS maps to produce the final CCRS favorability map of the study area.

### Composite common risk segment Map (CCRS)

**Fig. 9:**
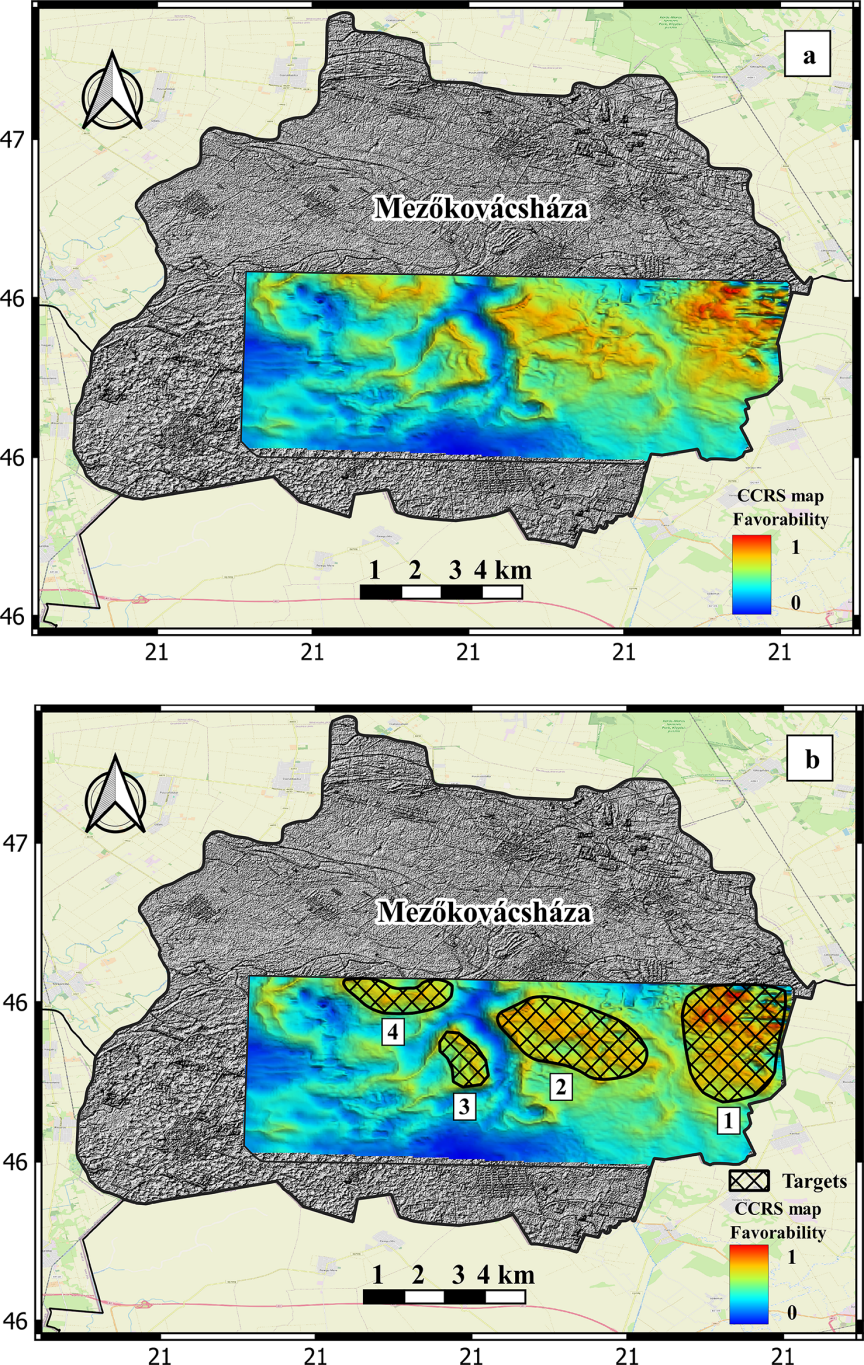
a) the CCRS favorability map of the study area. b) selected four areas as promising targets for geothermal exploration

The final product of the PFA approach is the normalized CCRS map which combine the CRS maps for the resource components; seal, reservoir fracture permeability, and heat source for the Mezőkovácsháza that reflects the risk associated with the geothermal resource exploration and identifies favorable resource tracks. Figure 9a shows the CCRS favorability map of the study area displaying varying favorability values.

The risk components (seal, fracture permeability, and heat source) have specific meanings where each plays a critical role in assessing the favorability of geothermal target areas. Their presence or absence directly affects the combined risk score, and their quantity is inversely correlated with the associated risk. The values between 0 and 1 represent a spectrum of risk and favorability. A value of 0 means that one, two, or all three of these components are missing, which signifies a high-risk area with low favorability. This is because even if two components are present, the absence of one (e.g., the heat source) can make the area nonviable for geothermal energy. Conversely, a value of 1 indicates the presence of all three components with optimal conditions, signifying low risk and high favorability. In this scenario, the geothermal system is expected to perform efficiently, making the area more attractive for development (Fig. 9b).

**Fig. 10:**
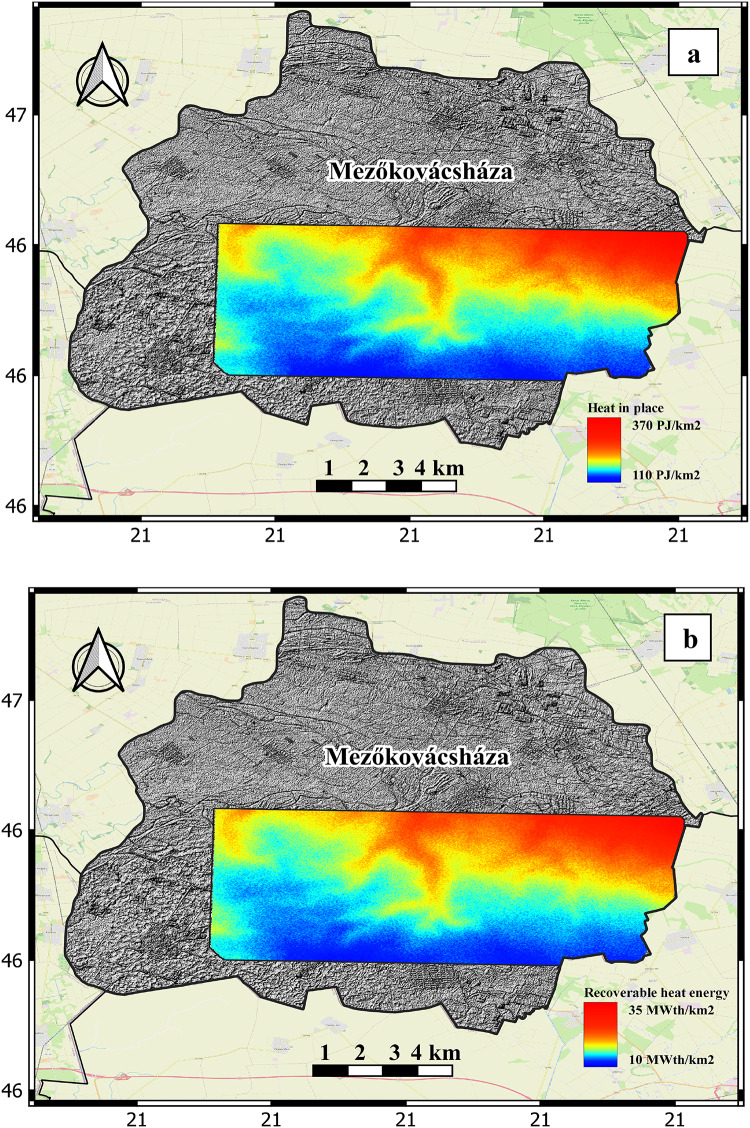
a) the estimated thermal energy reserves stored within the study area in PJ/km^2^2. b) the recoverable heat energy potential in MW_th_/km^2^2

By applying the same methodology used in successful global geothermal studies and projects globally (e.g^24–28^), four promising target areas are identified for future development within the study area. These targets were selected based on their favorable characteristics, aligning with established approaches that have proven effective in similar geological contexts worldwide (Fig. 10a).

### Geothermal potential for the selected areas

**Table 3:** The petrophysical parameters used to calculate the heat in place and the recoverable heat.

	Symbol	Property	Unit	Distribution	Values	Source
Petrophysical properties	φ	Porosity	fraction	Normal	0.05–0.15	^31^
	ρw	Fluid density	kg/m3	Normal	1040	^68^
	cpw	Fluid specific heat capacity	kJ/kg ◦C	Normal	4.8	^39^
	ρwr	Rock density	kg/m3	Normal	2500–2700	^31^
	Cr	Rock specific heat capacity	kJ/kg ◦C	Normal	0.9	^68^
Operational properties	Tref	Reinjection Temperature	◦C	-	30	^39^
	Rf	Recovery Factor	fraction	Normal	0.05–0.1	^39^
	Ce	Conversion Efficiency	fraction	-	0.85	^39^
	Pf	Plant Factor	fraction	-	0.95	^39^
	MPL	Mean Plant Lifetime	years	-	30	^39^

The four selected areas are assessed based on their area in Km^2^, stored energy in PJ, and recoverable Table 3 heat in MWth calculated based on the volumetric HIP method. Table 4 shows the amount of the total energy reserves stored for the selected areas.

**Table 4:** The amount of the total energy reserves stored for the four selected areas in PJ.

Location	Average stored energy (PJ/km^2^)	Area (km^2^)	Total stored energy (PJ)
1	195	53.8	10,491
2	210	39.1	8211
3	170	19.2	3264
4	200	12.7	2540

The amount of the total recoverable heat energy (Hrec) in MW_th_ that can be extracted from the estimated total reserves stored in the reservoir for the selected four areas is calculated for three production scenarios. For a conservative 30-year plan, a modest 20-year plan, and a fast 10-year plan (Table 5). From the geothermal calculation, the study area holds great geothermal potential that can be harnessed and used as energy source for many applications like district heating or even electricity generation for a small-town capacity.

**Table 5:** Total recoverable heat energy (Hrec) in MWth/year.

Location	Average Hrec MWth/km^2^	Area km^2^	Total Hrec MWth	Total Hrec MWth/year (30-year plan)	Total Hrec MWth/year (20-year plan)	Total Hrec MWth/year (10-year plan)
1	18	53.8	968	32	48	96.8
2	20	39.1	782	26	39	78.2
3	16	19.2	307	10	15	30.7
4	17.5	12.7	222	7.5	11	22.2

### Geothermal energy key limitations and challenges

Geothermal energy holds significant promise for contributing to a sustainable energy mix. Nonetheless, it is essential to recognize and address the inherent technical limitations and challenges that must be navigated to mitigate risks associated with geothermal development and production, ultimately facilitating a faster commercial adoption. Recent studies have examined the risks and challenges linked to the exploration and production of geothermal resources^67,68^. A study conducted by^67^, where The authors undertook a thorough compilation and standardization process to analyze and interpret the technical geoscientific and engineering challenges faced in individual geothermal projects. They refined the categories of key challenges into six main groups: infrastructure damage, productivity issues, health safety and environment, inadequate resources, injectivity problems, and drilling challenges.

Since 2011, Hungary witnessed a total of new 58 drilled thermal water wells^69^. The consistent rise in new wells annually illustrates the advancing geothermal sector in Hungary, attributable in part to the expansion of prior projects and in part to new district heating initiatives, alongside moderate growth in the agricultural sector. The growth of new reinjection wells represents a positive advancement, with a total of 13 reinjection wells drilled, primarily into permeable aquifers.

In addition to the universally shared challenges and risks inherent in geothermal exploration and development projects, such as the uncertainty of geological and structural models which, while not infallible, are nonetheless beneficial Hungary faces specific drilling challenges associated with deep, hard basement reservoirs, as elucidated by^48^. Furthermore, the environmental implications concerning the surface disposal of thermal wastewater are significant. Table 6 delineates the principal operational issues and challenges encountered in Hungary regarding geothermal exploration and development.

**Table 6:** Operational challenges encountered in Hungary regarding geothermal exploration and development.

Challenge	Explanation	Source
Reinjection	- Relatively simple into fractured-karstified carbonate reservoirs. - More complex into the Pannonian integranular reservoirs, as the necessary injection pressure can substantially increase within a relatively short time. - High clay content often cause the plugging of perforation in the well.	^71,72^
Dissolved gas content	- Thermal water with significant dissolved gas content (methane, nitrogen, CO2, H2S)	^71^
Scaling	- Carbonate scaling due to producing thermal waters with high dissolved content.	^71^
Economic Viability	- High upfront cost. - low historical prices have not incentivized the development of sufficient geothermal capacity.	^72,73^

In the current investigation, geothermal play fairway model (including cap or seal, reservoir fracture permeability, and heat source) is based on indirect geophysical characteristics or data which could be strong approach in the field. Nevertheless, certain limitations exist, as these components exhibit varying responses and sensitivities to other geophysical properties (e.g., resistivity, density, magnetic susceptibility). Future research could address these limitations by utilizing more comprehensive data and integrating additional geophysical field methods (such as electromagnetic, gravity, and magnetic techniques) to enhance the assessment of geothermal play fairway areas.

## Discussion

This study represents the first application of the PFA approach in geothermal exploration within the Mezőkovácsháza area and in all Hungary. Hungary’s strategic location in the geothermally active Pannonian Basin provides access to abundant geothermal resources, presenting a promising and sustainable alternative to conventional fossil fuels. The Mezőkovácsháza region, located in southeast Hungary, is particularly notable for its deep crystalline basement formations, which is found at about 1,000 m in depth and reach temperatures potentially exceeding 140 °C. These geological characteristics make the region an ideal area for geothermal energy exploration and development, offering significant potential for clean energy production. The investigation focused on three critical risk components essential for successful geothermal energy exploration in this play type: the heat source, reservoir fracture permeability, and seal integrity. These components were meticulously evaluated using the constructed geothermal PFA model, which is tailored specifically for the Mezőkovácsháza area. The PFA approach has been highly effective in the oil industry for years and has recently been adapted for geothermal exploration. This adaptation is demonstrated by Shervais et al. (2024), who successfully discovered previously undetected geothermal systems using PFA. Our study employs a similar strategy, but with a unique set of data, resulting in favorable outcomes and identifying promising prospects for geothermal development.

While the PFA in geothermal exploration has been increasingly popular in recent years, as evidenced by studies conducted by^24–28^, this study utilizes new input data for the first time in the PFA approach. These included colored inversion as an indication of fracturing, and high-resolution seismic attribute volumes. An essential aspect of this study involves incorporating advanced seismic techniques and well data to conduct a comprehensive evaluation of the subsurface geology. A comprehensive 3D post-stack seismic dataset, acquired in 2006 and processed in 2007, formed the cornerstone of the study. The seismic data interpretation process was conducted in two phases: conventional interpretation and attribute generation. The conventional interpretation phase focused on identifying major faults and defining the top boundaries of the seal and pre-Tertiary basement, providing a foundational understanding of the geological structures. This initial phase was crucial in establishing the structural framework of the subsurface, which is essential for understanding the potential for geothermal reservoirs.

The attribute generation phase applied various seismic attribute techniques, including frequency filtering attributes, structural attributes, and colored inversion. These advanced techniques provided deeper insights into the subsurface features, allowing for a more detailed understanding of the geological conditions that influence geothermal potential. The results from the seismic and well data interpretation, including generated attributes and the colored inversion cube, were integrated into the PFA framework using QGIS toolbox. This integration led to the creation of CRS maps for each resource component: heat source, reservoir fracture permeability, and seal integrity. Each CRS map represented the spatial distribution of risk associated with that particular component, highlighting areas with favorable geological conditions for geothermal development. By considering the combined risks associated with the heat source, reservoir permeability, and caprock integrity, the CCRS map is constructed and highlighted favorable resource areas with the highest potential for successful geothermal energy exploration and development.

Our findings are consistent with the work of^48^, who employed a GIS approach to identify potential areas for geothermal energy exploration in the Mezőkovácsháza region. Their proposed locations align closely with those identified in our study, validating our PFA model and demonstrating the effectiveness of the PFA approach, particularly in exploring blind geothermal systems. As a further step, we quantified the geothermal capacity of the chosen targets, providing a more comprehensive quantitative assessment of the area’s geothermal potential. This additional calculation confirmed the robustness of our methodology, particularly in terms of its applicability to blind geothermal systems where surface manifestations are not readily visible. Through utilizing the PFA and conducting a thorough assessment of key risk factors associated with a functional geothermal system such as heat, reservoir fracture permeability, and seal capacity, this study successfully narrowed down an expansive exploration area of around 350 km^2^ to just 4 highly promising target sites (Fig. 9b). These prospects indicate a convergence of critical risk factors with a low-risk assessment and high favorability.

The careful examination of the CRS map of reservoir fracture permeability suggests that the reservoir fracture permeability in the study area poses a notable risk factor for geothermal exploration and development. The permeability of the reservoir fractures, unlike the seal and heat source components, is not uniformly distributed and exhibits significant variation as it pertains to fracture-based permeability. In order to effectively minimize potential risks, it is crucial to investigate and measure the reservoir fracture permeability with precision.

## Conclusion

The application of the PFA approach, when utilized with GIS tools and advanced seismic attribute analysis, has resulted in noteworthy discoveries in the Mezőkovácsháza region. The generation of CRS maps for each critical risk component within the study area, including heat source, reservoir fracture permeability, and seal integrity, in addition to the ultimate favorability CCRS map, offers a robust tool for the identification of promising geothermal prospects.

The geothermal PFA model of the Mezőkovácsháza study area has been constructed, and several inferences have been derived from it, which are outlined below:

- The study area exhibits considerable potential as a source of clean energy for direct use applications and electricity generation.
- The study area poses varying risk levels associated with geothermal exploration and development.
- The successful process of conventional geothermal exploration and development in the study area relies heavily on the presence of the three key resource components. Lacking even one of them, the process would come to a halt.
- The weights of the three resource components are not equal as the fact that they do not hold equal risk.
- The reservoir fracture permeability is the main risk factor, and it plays a crucial role in determining the success of the geothermal exploration and development. Accurately measuring and having confidence in this factor is of utmost importance.
- The study area exhibits substantial potential for the deployment of Enhanced Geothermal Systems (EGS), particularly in areas where natural fracture components are absent. This is due to the area’s highly favorable conditions for heat source and seal components.

The study finding is not only exciting from a resource exploration standpoint but also holds immense practical value for sustainable energy development in Hungary. The quantification of geothermal potential included estimates of total thermal energy reserves stored within the study area, measured in petajoules (PJ), and the recoverable heat energy in terms of megawatts thermal (MW_th_) under various production scenarios, including a conservative 30-year plan, a modest 20-year plan, and a fast 10-year plan. These scenarios provided a range of options for potential geothermal energy development, allowing for flexibility in planning and decision-making.

For future work, a techno-economic model will be used to assess the practicality and financial considerations of drilling geothermal doublets and building a geothermal power plant. This analysis will provide valuable information on the financial feasibility of geothermal energy projects in the region.

Overall, this study stands out for its innovative and thorough approach to geothermal exploration risk assessment. The combination of advanced seismic attribute analysis and the PFA framework offers a more comprehensive and data-driven methodology for identifying promising geothermal prospects. The study not only advances geothermal exploration methodologies but also provides valuable insights for informed decision-making in sustainable energy development, not just in the Mezőkovácsháza study area but throughout Hungary.

## Electronic supplementary material

Below is the link to the electronic supplementary material.

Supplementary Material 1

## Data Availability

The datasets utilized and/or analyzed during the current study are available upon request from the corresponding author.
