# Supplementary material for: Innovative method integrates play fairway analysis supported with GIS and seismic modeling for geothermal potential evaluation in a basement reservoir
Source: Sci Rep. 2025 Jan 8;15:1325. doi: 10.1038/s41598-024-79943-6 (PMC11711507; doi:10.1038/s41598-024-79943-6)

**Innovative Method Integrates Play Fairway Analysis supported with GIS and Seismic modeling for Geothermal Potential evaluation in a Basement Reservoir**

**Figure 1S.** An example of the seismic to well tie result for Domb_DNY_6 well. The tie shows a high cross correlation of 83% between the seismic data and the synthetic seismogram.


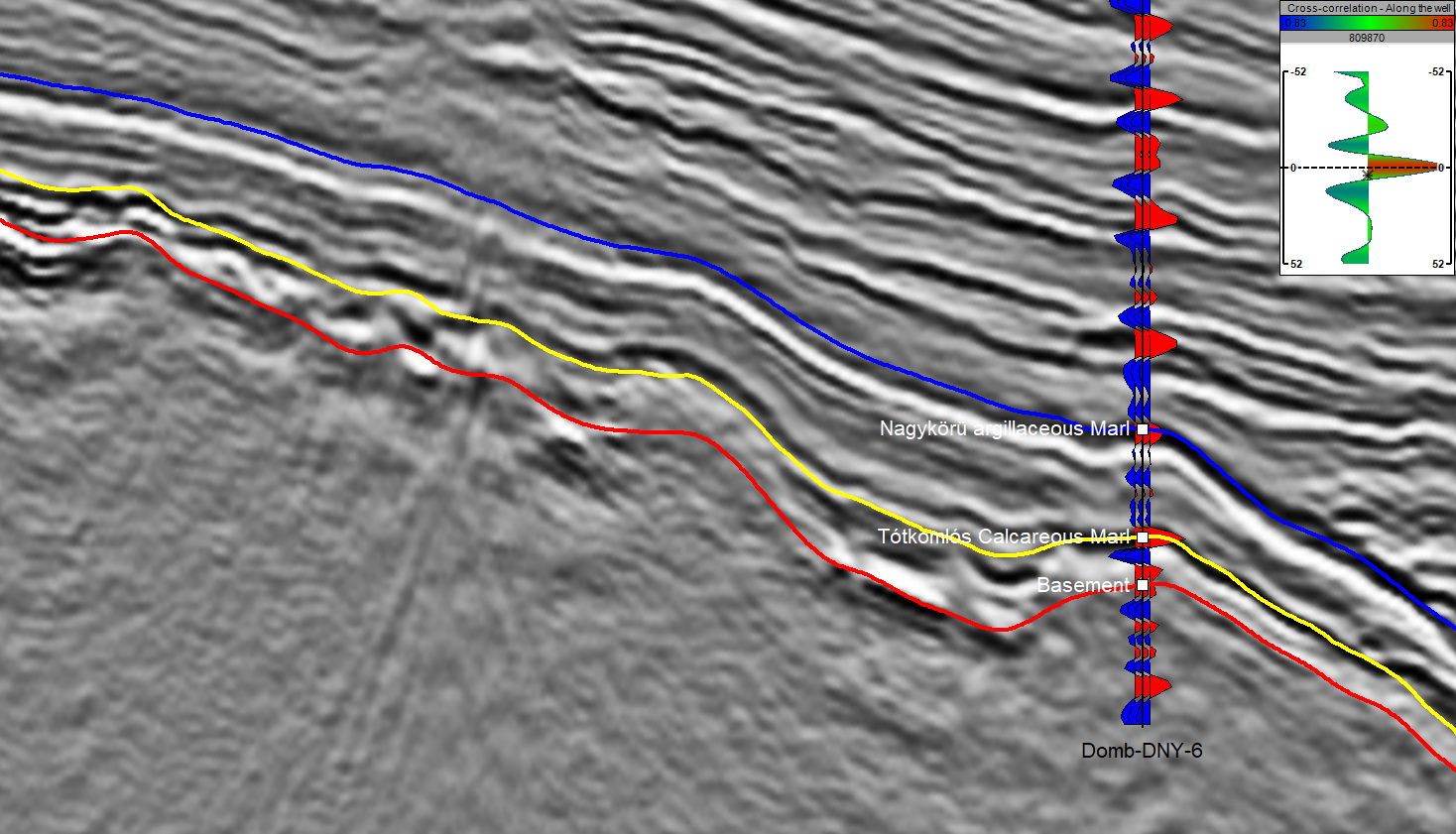

Supplement: Supplementary file 1 — Supplementary Material 1 [file 41598_2024_79943_MOESM1_ESM.docx]
